# Rich Club Organization of Macaque Cerebral Cortex and Its Role in Network Communication

**DOI:** 10.1371/journal.pone.0046497

**Published:** 2012-09-28

**Authors:** Logan Harriger, Martijn P. van den Heuvel, Olaf Sporns

**Affiliations:** 1 Department of Psychological and Brain Sciences, Indiana University, Bloomington, Indiana, United States of America; 2 Department of Psychiatry, University Medical Center Utrecht and Rudolf Magnus Institute of Neuroscience, Utrecht, The Netherlands; Newcastle University, United Kingdom

## Abstract

Graph-theoretical analysis of brain connectivity data has revealed significant features of brain network organization across a range of species. Consistently, large-scale anatomical networks exhibit highly nonrandom attributes including an efficient small world modular architecture, with distinct network communities that are interlinked by hub regions. The functional importance of hubs motivates a closer examination of their mutual interconnections, specifically to examine the hypothesis that hub regions are more densely linked than expected based on their degree alone, i.e. forming a central rich club. Extending recent findings of rich club topology in the cat and human brain, this report presents evidence for the existence of rich club organization in the cerebral cortex of a non-human primate, the macaque monkey, based on a connectivity data set representing a collation of numerous tract tracing studies. Rich club regions comprise portions of prefrontal, parietal, temporal and insular cortex and are widely distributed across network communities. An analysis of network motifs reveals that rich club regions tend to form star-like configurations, indicative of their central embedding within sets of nodes. In addition, rich club nodes and edges participate in a large number of short paths across the network, and thus contribute disproportionately to global communication. As rich club regions tend to attract and disperse communication paths, many of the paths follow a characteristic pattern of first increasing and then decreasing node degree. Finally, the existence of non-reciprocal projections imposes a net directional flow of paths into and out of the rich club, with some regions preferentially attracting and others dispersing signals. Overall, the demonstration of rich club organization in a non-human primate contributes to our understanding of the network principles underlying neural connectivity in the mammalian brain, and further supports the hypothesis that rich club regions and connections have a central role in global brain communication.

## Introduction

Large-scale networks of anatomically defined brain regions and their interconnecting pathways can be represented in the form of connection matrices and statistically analyzed as graphs [1]–[6]. Previous studies of mammalian cortex and whole-brain connectivity data sets revealed several characteristic features such as high levels of clustering and a short path length [7]–[9], structurally distinct network communities (clusters or modules) consisting of functionally related brain regions [7], [10]–[13], cost-efficient wiring [14], [15], and highly connected and highly central hub regions [16]–[19]. From a functional perspective, modules and hubs are of particular interest. Modules comprise communities of brain regions that are not only mutually connected but also share many afferent and efferent projections, and are thus likely to exhibit similar physiological responses and functional specialization [20]. Given a modular architecture, hubs are critical for integrating neural signals across communities and thus for ensuring coherent information processing across the network.

A number of network studies have identified examples of networks where hub nodes are densely interconnected, beyond what would be predicted based on their large number of connections (high degree) alone, giving rise to the so-called “rich club” phenomenon [21]. A neuronal rich club was first detected in the network of interregional projections of the cat cortex obtained from tract tracing studies [22], and later seen also in human whole-brain and cortical connectivity derived from non-invasive diffusion imaging and tractography [23], [24]. Now collations of anatomical tract tracing data from the macaque monkey ([25]–[27]) provide an opportunity to investigate rich club organization in a non-human primate. Importantly, tract tracing data provides information on the direction of projections and thus allows insights into the anatomical relations between a putative rich club and the rest of the brain that cannot be obtained from present-day diffusion imaging/tractography. This includes an analysis of characteristic network motifs, the direction of information flow along paths, and insights into how these communication patterns relate to structural modules and the anatomical locations of rich club and other regions of the macaque brain.

This report builds on a recently published collation and network analysis of macaque connectivity data [27] as well as on reports on rich club organization in cat cortex [22] and human brain [23], [24]. Extending this earlier work, we provide evidence for the existence of rich club organization in a non-human primate, map the spatial extent and topology of the rich club across the brain, determine the relation of rich club regions to structural network communities, identify significant network motifs that are aggregated around rich club regions, and estimate the degree to which macaque rich club regions and connections contribute to short paths across the network. We compare our results to those of other studies that have demonstrated rich club organization in other species, including humans, and hypothesize that rich club organization is of central importance for global brain communication.

## Methods

### Connectivity Data

The connectivity data set used in this study comprises anatomical data from 410 tract tracing studies collated in the online neuroinformatics data base CoCoMac (http://cocomac.org; http://cocomac.g-node.org; [28], [29]), first analyzed and made publicly available by Dharmendra Modha and Raghavendra Singh [27]. In the present study we focused primarily on an analysis of the connectivity among regions of the cerebral cortex, in order to focus on cortical processing in the context of earlier studies in other species (e.g. [16], [22]–[24]). The cortical connection matrix was extracted from the primary connection data by removing all subcortical (thalamus, basal ganglia, brainstem) regions. In addition, regions that did not maintain at least one incoming and one outgoing connection were also removed to ensure that the network was fully connected. The remaining connection data set used in this study consisted of 242 regions and 4090 directed projections represented in binary format (connection present = 1, connection absent = 0). No distinction was made between strong versus weak connections, or between connections that were shown to be absent versus those for which no data were available. The matrix contained no self-connections. The connectivity matrix together with a list of region names is included in the Supporting Information (File S1 and File S2). A whole-brain connection matrix was also generated, following the same procedure described in this section. It included all subcortical regions and contained a total of 352 regions and 6491 directed edges.

### Graph Measures

Most graph-theoretical analyses reported in this paper were carried out using the publicly available Brain Connectivity Toolbox (www.brain-connectivity-toolbox.net; [30]).

#### Small-world metrics

Clustering coefficients, path lengths, and local and global efficiency were computed following standard protocols, using computational tools appropriate for directed binary graphs (see e.g. [5], [30]). Clustering coefficients and path lengths for the cortical network were derived from the clustering coefficients of individual regions, and from the network's distance matrix (excluding circular paths), respectively. Both measures were scaled by comparison with populations of randomized and latticized networks, respectively representing two classes of null models (see below) to derive the normalized clustering coefficient γ and the normalized path length λ. Two metrics were employed to express the “small-worldness” of the network. The small world index σ [31] was computed as the ratio of the normalized clustering coefficient and the normalized path length. Values of the index that are greater than 1 indicate the presence of high clustering and a short path length, both compared to only randomized null models. In addition, we computed a recently proposed alternative metric ω [32] that compares clustering and path length to latticized and randomized networks, respectively. A value of ω near 0 indicates the presence of small-world attributes, i.e. clustering similar to lattice networks and path lengths similar to random networks.

#### Modularity

The community structure of the network was identified by optimizing a network-based modularity metric that takes into account the direction of network edges [33]. The metric belongs to a family of modularity measures (e.g. [34]) that quantifies the density of edges present within communities (given a network partition) relative to what would be expected if edges were distributed uniformly. A spectral optimization technique was applied to identify the partition for which the modularity metric was maximized.

#### Centrality Measures

Numerous graph-based measures for determining the centrality or influence of individual nodes have been proposed. Here, we selected two measures based on paths and topological distances, and two measures based on the effects of perturbations (node deletion) on the network. Betweenness centrality quantifies the fraction of shortest paths between all node pairs that travel through a given node. Closeness centrality is the average distance between a given node and all other nodes in the network, and is expressed here as the average of the in-closeness and the out-closeness. Vulnerability [35] is computed as the decrease in global efficiency due to the deletion of a given node. Dynamical importance [36] is a spectral graph measure designed to capture the contribution of a given node to system dynamics, expressed as the decrease in the magnitude of the largest eigenvector of the adjacency matrix after the node is deleted. In addition to these centrality measures, the participation coefficients of each node, given a network partition that optimizes modularity, were calculated [37]. The participation coefficient was used to identify connector hubs, defined as nodes with high degree (within-module z-score>2) and diverse connectivity (participation coefficient >0.5).

#### Motifs

Networks can be uniquely decomposed into small connected subgraphs called motifs [38], each consisting of a fixed number of nodes. In directed networks, 13 connected motifs among 3 nodes are possible, and the frequency with which these motifs occur in a given network can provide information about local connectivity patterns that are functionally important. To control for the effects of network density and degree sequence on empirically observed motif frequencies it is necessary to compare these frequencies against suitable null models (see below). In the present study, motif frequencies for 3-node motifs in the macaque cortical network and populations of null models were derived using standard approaches [38], [39]. Additionally, a heuristic search algorithm was employed to identify, for each network node, the largest star motif, defined as a subgraph consisting of a central node that is reciprocally connected to otherwise mutually unconnected nodes.

#### Rich Club Detection

**Figure 1:**
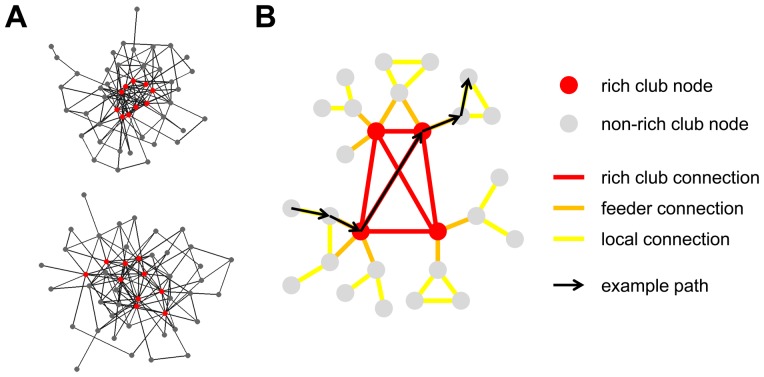
Rich club detection and node/edge classification. (A) Example networks, each composed of 48 nodes and 123 undirected edges, and displayed using a spring-embedding layout algorithm. Both networks contain a set of high-degree nodes (red). In the network at the top, these nodes are interconnected with a density (rich-club coefficient) of 0.711. The network below was derived by randomizing the original network, preserving all node degrees. High-degree nodes are again shown in red, and their connection density is 0.578. Comparison of the network at the top to a population of 10,000 randomized networks indicates the presence of a rich club, with p = 0.0004. Note that due to constraints imposed by the degree sequence high-degree nodes in the randomized network remain linked with an edge density that exceeds that of the network as a whole. (B) Identification of rich club nodes allows the classification of edges into rich club, feeder and local edges.

A rich club is defined as a set of high-degree nodes that is more densely interconnected than predicted on the basis of the node degrees alone ([21]; Figure 1A). Here, we take node degree *k* to stand for the sum of each node's in-degree and out-degree. Rich club (RC) detection involved the following steps. (1) For a given degree *k*, all the nodes with a degree ≤*k* were removed. (2) The rich-club coefficient *Φ(k)* was computed as the ratio of the remaining connections over all possible (essentially, *Φ(k)* is equal to the connection density of the subgraph of nodes with degree *>k*). (3) For the same set of nodes, the subgraph density for nodes with degree *>k* was computed for a population of 10,000 randomized networks preserving degree sequence (see below). (4) The normalized rich-club coefficient *Φ_norm_(k)* = *Φ(k)*/*Φ_random_(k)* was derived, with *Φ_random_(k)* representing the average rich club coefficient over all 10,000 randomized networks. (5) Steps 1–4 were repeated from the lowest degree to the second highest degree encountered in the macaque network, i.e. ranging from *k = 2* to *k = 121*. A rich club is potentially present if *Φ_norm_* is >1 over a range of *k*. (6) To assess statistical significance, the null distribution of 10,000 rich-club coefficients obtained from the randomized networks was compared to the empirical value *Φ(k)* for the macaque network at each level *k*, by first deriving a one-sided p-value. Then, to correct for multiple comparisons over the range of degrees *k* examined, false-discovery rate correction was performed [40], limiting the false discovery rate to 0.05. (7) In many networks, including the network under study here, *Φ_norm_* is >1 and statistical comparison to the null model reaches significance for several different values of *k*, indicating the presence of rich club organization in the network. Going over the range of values of k, unique sets of nodes (and their corresponding subgraphs) were extracted. These sets could be arranged in a nested sequence ranging from the set of rich club nodes with the highest degree to the one with the lowest degree. The order of this sequence corresponded to the rich club level, with nodes at level 1 forming a subset of nodes at level 2, and so forth.

#### Node and Edge Classification

Once a rich club has been identified, network nodes can be classified (Figure 1B) as rich club nodes and non-rich club nodes, and network edges can be classified as linking rich club nodes amongst each other (rich club connections), rich club nodes to non-rich club nodes (feeder connections), and non-rich club nodes amongst each other (local connections) [24]. This classification was used in several analyses to highlight different contributions of node and edge classes to various graph-theoretical metrics.

### Null Models

For statistical assessment of graph topology two different null models were implemented. The first null model involved randomization of edges using a Markov random switching algorithm [41] that preserved network size, density, and the in-degree and out-degree sequence. The resulting population of randomized networks thus preserved local node statistics (most importantly, each node's in- and out-degree) while losing global network structure. The second null model used the same randomization algorithm but imposed the additional constraint that upon rewiring edges must move closer to the main diagonal of the connection matrix, thus maximizing connection density among “neighboring” nodes. This “latticization” model represents a second null hypothesis that embodies cost-efficient wiring in an approximate ring lattice. For each instantiation of the lattice model, a different randomly assigned node ordering was imposed.

It should be noted that randomization of networks is subject to the constraints imposed by the degree sequence. These constraints can make the construction of randomized networks difficult or impossible, particularly in dense networks containing a mixture of high-degree and low-degree nodes, as encountered in rich club architectures [42]. In the case of the macaque cortical connectivity matrix, the low density of the network did allow a sufficiently large reconfiguration space for robust rich club detection by comparison to randomized populations.

### Spatial Embedding and Cost Analysis

The original data set [27] did not contain spatial information on the position and extent of brain regions and/or projections. To estimate approximate spatial positions of cortical regions, we followed an approach introduced in previous studies of macaque cortex spatial embedding and wiring cost [14]. Regions contained in the 242-node connection matrix were matched to macaque cortex surface coordinates reported in the SumsDB data base (http://sumsdb.wustl.edu/sums; [43]). Matches were performed manually, on the basis of correspondences of regions to five major areal parcellation schemes and atlases [44]–[49], followed by consultation of other parcellation schemes to establish additional correspondences among remaining unmatched regions. Overall, 187 regions maintaining a total of 3199 projections could be matched to surface coordinates in SumsDB. The 30,736 vertices of a macaque cortex surface mesh representing the right cortical hemisphere were “painted” with regional assignments as established in the matching process. As a result over 90 percent of the macaque surface mesh could be assigned to at least one of 187 cortical areas. Note that since the set of regions collated by Modha and Singh does not represent a single unique parcellation, and since numerous areas were assigned multiple overlapping sets of surface placement data on the basis of their inclusion in several parcellations, coverage of the macaque surface was not complete, and in areas where parcellations disagree some of the surface vertices were labeled with more than one region name. In case of conflicting assignments, vertices were given unique assignments by majority vote. The resulting surface mesh was used for creating graphical renderings of network metrics on an inflated surface representation.

Once placement of regions on the macaque surface mesh was accomplished, center-of-mass spatial coordinates for the non-inflated macaque surface were computed for each region, as the average of the spatial coordinates of all of the region's constituent vertices. These regional center-of-mass coordinates were then used for deriving Euclidean distances to estimate the lengths of inter-regional projections. Since anatomical fiber pathways often follow curved trajectories, these Euclidean distances are likely underestimating the actual lengths of axonal projections approximating a lower bound on physical wiring length. Euclidean distances as estimates of connection lengths entered into a measure of communication cost per edge, defined previously [24] as the number of times the edge appears on a short path multiplied by its physical length. Note that Euclidean distances between regions are entirely separate from their topological distances (length of the shortest path), determined on the basis of their network connectivity.

## Results

Unlike the whole-brain matrix of Modha and Singh [27], the connection matrix used in this paper comprises only regions of cerebral cortex. Hence, we begin by examining several global graph metrics, determining the network's community structure, and identifying putative hub regions. We then move to an analysis of rich club organization, including its contributions to motifs, directed short paths, and aspects of spatial embedding and communication cost.

### Global Graph Metrics

Graph-theoretical analysis confirmed several characteristic architectural properties reported previously in other data sets (e.g. [7]–[9], [16]), as well as in the original study [27] which introduced the data set used in the present paper. Macaque cortex exhibited highly clustered connectivity (γ = 2.072) while maintaining a short path length (λ = 1.058), resulting in a small-world index σ = 1.959. Comparison of clustering and path length to both randomized and latticized null models yielded a value for the small-world metric ω = 0.404 that is consistent with the presence of small-world attributes [32]. The macaque cortical network exhibited high global efficiency (0.443, comparable in magnitude to a mean of 0.463 for a population of 1000 random networks) and high local efficiency (0.565, significantly greater than a mean of 0.356 in random networks), again consistent with the presence of a small-world architecture [50].

### Modularity

**Figure 2:**
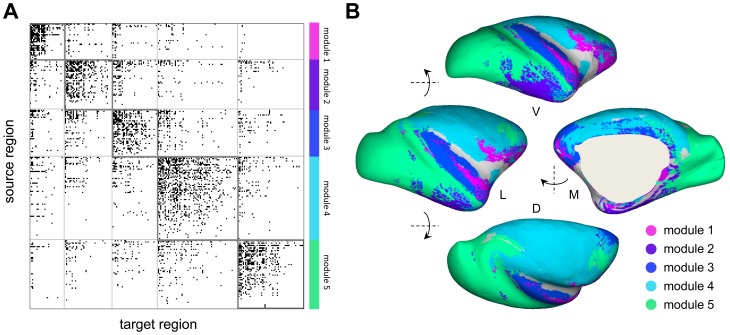
Connection matrix and network communities (modules). (A) Binary connection matrix comprising 242 regions and 4090 directed projections. Regions are arranged by module assignment, and within each module they are ranked by their degree (sum of in-degree and out-degree). The node ordering and module assignments are reported in the Supplementary Information. (B) Surface rendering of the inflated right hemisphere of macaque cortex, with surface regions color-coded by to their module assignments. Some points on the surface corresponded to multiple cortical regions since multiple schemes for regional parcellations were used for surface mapping. This could lead to multiple module assignment for a given surface point. In case of multiple assignments, regions were colored according to a majority rule, choosing the mode of the distribution of module assignments. In the case of a tie, a module was chosen at random from the tied set, resulting in a mottled appearance. Surface plots at left and right show lateral (L) and medial (M) views; plots at top and bottom show ventral (V) and dorsal (D) views.

Optimization of a network-based modularity metric [33] resulted in a partition of the network into 5 structural modules, with a value of the modularity measure Q = 0.362. Intra-modular edges accounted for 58% of all connections, resulting in an average within-module connection density of 0.191 versus a between-module density of 0.037. Figure 2A displays the macaque cortex connection matrix, with the regions rearranged to show the individual modules. In addition, within each module, nodes are ranked according to their degree (sum of in-degree and out-degree). This node arrangement shows that regions of macaque cortex exhibit a broad distribution of node degrees, previously characterized as exponential [27], and that high-degree nodes are encountered in each of the 5 modules. A rendering of the spatial layout of these modules on an inflated surface plot of the macaque cortex is shown in Figure 2B. Structural modules mainly consist of spatially contiguous regions comprising frontal/orbitofrontal (“module 1”), inferior temporal (“module 2”), frontal/superior temporal (“module 3”), prefrontal/motor/somatosensory (“module 4”), and occipital/visual/prefrontal regions (“module 5”).

### Centrality and Hubs

**Figure 3:**
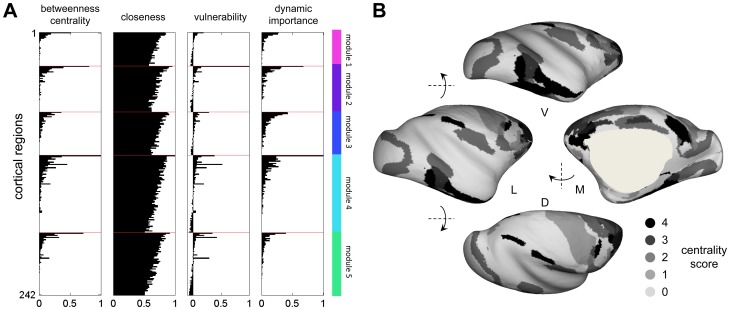
Centrality and hub regions. (A) Regional scores for node betweenness, closeness, vulnerability and dynamic importance. Regions are arranged in the same order as shown in Figure 2A. Module assignments (see Figure 2) are indicated by the color bar at the right. (B) Surface rendering of the global centrality score.

The broad spatial distribution of highly central network elements across all modules was further substantiated by an analysis of several centrality and influence measures, two of them based on short communication paths (node betweenness centrality and closeness centrality) and two based on network perturbation (vulnerability and dynamic importance; Figure 3A). As expected, all four centrality measures were highly correlated with node degree. Assessing the consistency with which nodes were placed in the top 10% for each centrality measure allowed the construction of a global centrality score [16],[19],[51]. In line with previous analyses [27] a set of 13 top-ranked regions, found in the 90^th^ percentile across all four centrality measures, was identified, including areas 13a (module 1), 24, TF, TH (module 2), 32, 11, TE (module 3), 46, 7b, 23c, PGm (module 4), LIP and 8A (module 5). Figure 3B shows the spatial layout of the global centrality score, demonstrating that putative hub regions are widely distributed across the surface of the macaque cortex. The magnitude of the nodal participation coefficient (here >0.5), in conjunction with each node's within-module z-score for node degree (here >2), allowed the classification of 12 connector hubs (regions 13a, 12o, 12l, 24, TF, TH, 32, 9, 46, LIP, 8A, and PIT). At least one connector was found within each of the 5 structural modules.

### Rich Club Detection

**Figure 4:**
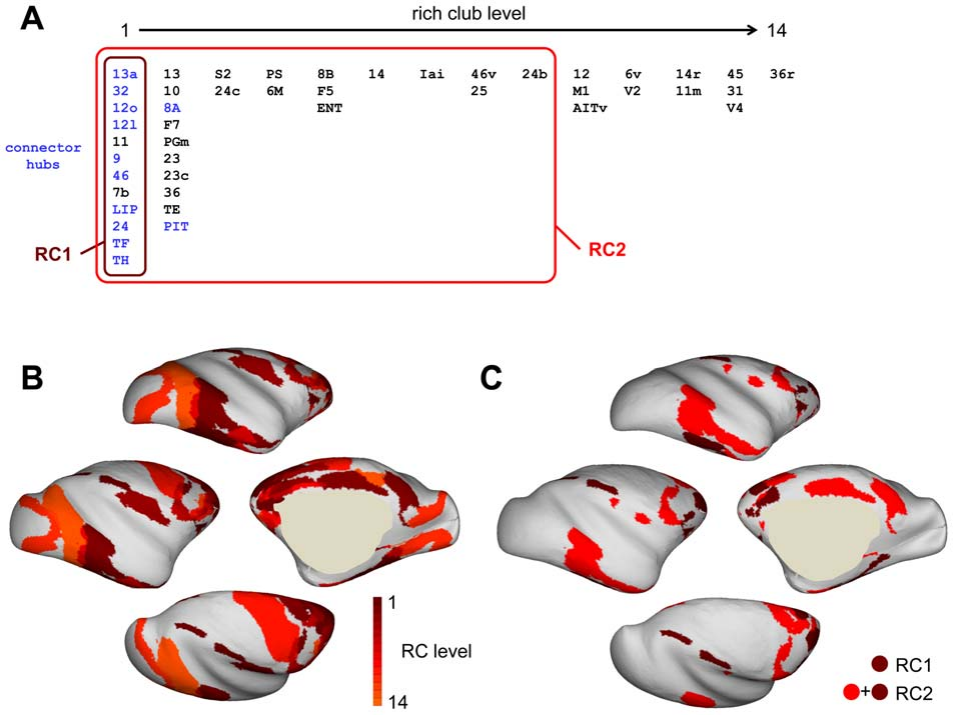
Rich club regions and their spatial distribution. (A) List of rich club member regions across all 14 rich club levels. Member regions of RC1 and RC2 are indicated, as are connector hubs (listed in blue). (B) Surface map of RC levels. (C) Surface maps of RC1 and RC2 member regions.

The broad spatial distribution of hub regions might suggest that these regions are largely disconnected, linked primarily to other members of their own network community. However this was not found to be the case. Instead, examining the density of interconnections among macaque cortical regions relative to random null distributions demonstrated the existence of a rich club (RC) across an extended range of node degrees. Rich club coefficients were computed for the macaque cortex as well as a population of 10,000 randomized networks (preserving network size, edge density, and in- and out-degree sequence). After statistical analysis and performing a false-discovery-rate correction [40], 14 unique sets of nodes were detected. These 14 sets were arranged into a nested hierarchy corresponding to 14 rich club levels. From rich club level 1 (innermost) to 14 (outermost), the RCs ranged in size from 12 to 45 nodes, and all formed fully connected components. The level at which each region became a member of the rich club is shown in Figure 4A.

Two RCs were selected for more detailed analyses, one of them constituting the tightest, most densely connected RC (RC1; FDR-corrected p = 0.045, *n* = 12, *Φ(k)* = 0.64) and the other representing the rich club that survived statistical testing with the lowest corrected p-value (RC2; FDR-corrected p = 0.008, *n* = 34, *Φ(k)* = 0.43). Members of RC1 consisted of prefrontal areas 13a, 12o, 12l, 11, 9 and 46; paracingulate area 32, anterior cingulate area 24; parietal areas 7b and LIP; and temporal areas TH and TF. In addition to all regions contained in RC1, members of RC2 included frontal/prefrontal areas 8A, 8B, F5, F7, 10, 13, 14, PS, 46v, 25, and 6M; cingulate areas 23, 23c, 24b, and 24c; parietal area PGm; temporal areas TE and PIT; perirhinal area 36 and the entorhinal cortex ENT; as well as agranular insular cortex Iai. Both, RC1 and RC2 contained at least one region from each of the 5 structural modules. Figure 4C shows the spatial locations of RC1 and RC2 regions on an inflated surface plot of the macaque cortex. RC2 occupies portions of medial and superior parietal cortex, prefrontal and orbitofrontal cortex, anterior and posterior cingulate cortex, and inferior temporal cortex. RC1 consists of a smaller, yet spatially widely distributed set of regions, involving parts of parietal, frontal, temporal and cingulate cortex.

Following an identical procedure for rich club detection in the 351 region whole-brain connection matrix again resulted in several nested sets of regions that were found to be more strongly interconnected than equivalent random null models (a population of 5,000 randomized networks that preserved degree sequence). The innermost whole-brain rich club level consisted of 10 cortical regions (13, 13a, 32, 12o, 11, 9, 46, LIP, 24, TF), all of them also found at the innermost 2 levels of the rich club detected in the 242-node macaque cortical matrix (Figure 4A). With the exception of region MD (the mediodorsal nucleus of the thalamus) no subcortical regions became members of the whole-brain rich club at higher rich club levels.

### Rich Club and the Network Core

**Figure 5:**
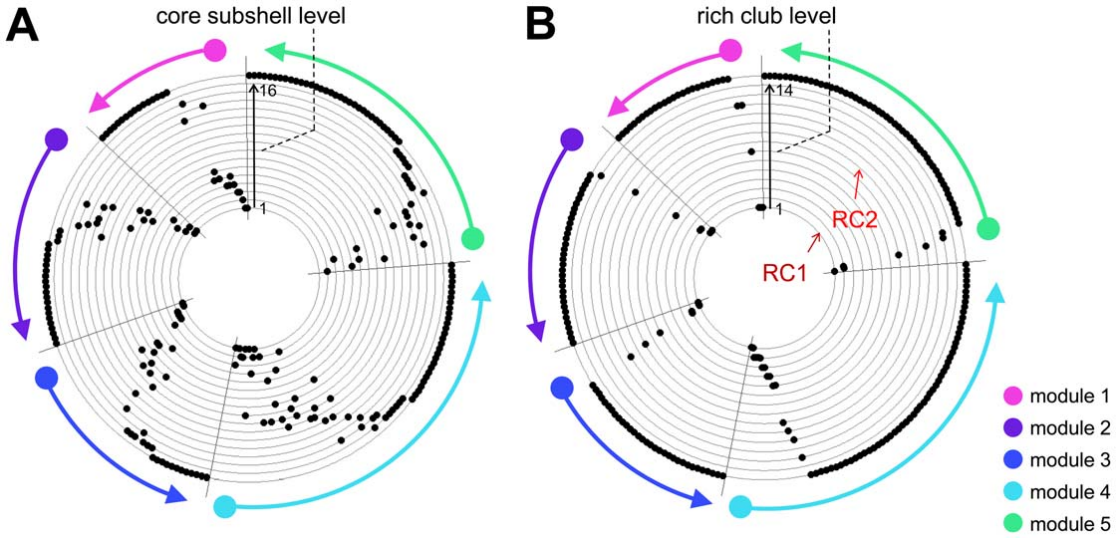
Comparison of core subshells and rich club members. In both plots, the nodes are arranged along a circle, in the same order (by module and degree) used for displaying the connection matrix in Figure 2A, starting counterclockwise at the top. (A) Core subshell levels are displayed as concentric rings, with the innermost subshell nearest to the center of the circle. Regions are marked as black dots according to their subshell level. (B) Rich club levels are displayed as concentric rings, with the tightest rich club (RC1) corresponding to rich club level 1. Regions are marked as black dots according to their rich club level.

Another approach towards identifying highly connected subgraphs is to perform k-core decomposition of the connection matrix, a procedure that recursively removes low-degree network elements to reveal subgraphs consisting of high-degree nodes [17], [52]. The highest-degree (*k = 25*) subgraph surviving k-core decomposition comprised 104 nodes, including all of the regions contained in the RCs. At *k = 26* the network fully disintegrated and no connected core or subgraph was retained. In analogy to the subshell level defined by Modha and Singh [27], the order in which nodes detached from the network at *k = 26* provided information about the degree to which they were coherently connected. Figure 5A shows a plot of the subshell level (concentric circles) for all nodes, arranged by module and node degree, and displayed counter-clockwise in the same order as in the connection matrix in Figure 2A. For comparison, Figure 5B shows a corresponding plot of the membership of all nodes in the 14 unique RCs (again arranged concentrically), with nodes displayed in the same ordering scheme as in Figure 5A. Comparison of Figure 5A and 5B revealed similarities in k-core decomposition and rich-club membership, and across all rich club nodes the corresponding subshell levels and rich club levels were significantly correlated (r = 0.556, p<0.001). An overlapping set of high-degree nodes occupied the innermost core subshell levels as well as the innermost rich-club layers. Notably, the most compact RCs as well as the two innermost subshells contained members of each of the five structural modules. Hence, all five modules contributed to the nodes and edges forming the densest parts of the network core and the rich club.

### Rich Club Motifs

The identification of characteristic structural motifs requires the definition of suitable null models for statistical comparison. Here, as in earlier work [39], we chose to define two null models implementing hypotheses of “no global connectivity structure” (randomized networks), as well as “cost-optimal placement of network nodes and edges in a ring lattice” (latticized networks), both representing the extreme boundaries of the classic small-world model [53]. The proportion of reciprocal connections (defining a 2-node motif of two mutually connected nodes) encountered in the macaque cortex (0.507) was intermediate between that of randomized (0.148±0.006) and latticized populations (0.599±0.014).

**Figure 6:**
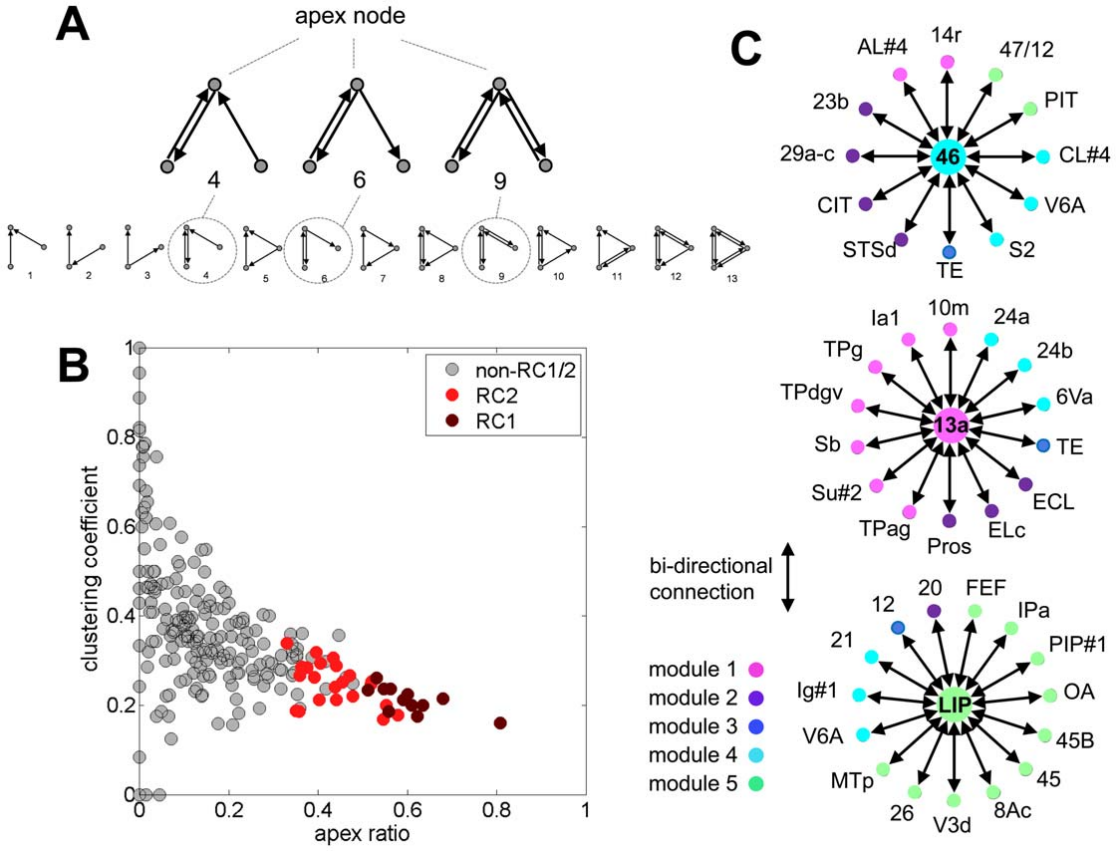
Motif contributions of rich club regions. (A) Apex motifs 4, 6 and 9 are statistically overrepresented as compared to both randomized and latticized null models (z-scores 13.1, 12.3, and 59.5 against randomized controls; 15.1, 16.9 and 2.2 against latticized controls). All 13 3-node motifs are shown at the bottom. (B) Scatter plot of apex ratio (for all three apex motifs) and clustering coefficient for all 242 regions, with RC1 and RC2 color coded. (C) Examples of star-like motifs centered on rich club regions 46, 13a, and LIP. All surrounding regions are reciprocally connected to the center and unconnected otherwise. Regions are color coded by their module assignment.

The matrix of macaque cortical connectivity was decomposed into 3-node motifs and their motif frequencies were compared to those encountered in both randomized and latticized networks. Three out of thirteen motif classes were found to be statistically over-represented in macaque cortex. These three motifs each consisted of a central or apex node that is either bi-directionally (motif 9) or uni-directionally linked to two mutually unconnected nodes (Figure 6A). Each region of macaque cortex participated in a subset of these motifs, with the proportion of motifs where a given node occupied the motif's apex expressed as the apex ratio [16]. Rich club nodes contributed to a large fraction of apex motifs, with a high proportion of instances where the rich club node occupied the apex position, resulting in a high apex ratio. For example, the 12 nodes comprising RC1 are found at the apex of 4554 instances of motif 9, out of a total of 9574 instances found in the macaque cortex (RC2: 7019 out of 9574 instances). The average apex ratio for motifs of class M9 is 0.59 for RC1 and 0.45 for RC2, compared to 0.12 for non-RC1 and 0.10 for non-RC2 nodes (both p<0.001). Because of the topology of the three apex motifs, a high apex ratio of a given node should tend to lower its clustering coefficient, as the apex node joins otherwise unconnected node pairs. Figure 6B shows the relationship between the apex ratio (averaged across all three apex motifs) and the clustering coefficient, with RC1 and RC2 nodes exhibiting markedly high apex ratios and low clustering coefficients, relative to non-RC nodes.

The aggregation of apex motifs around RC nodes gave rise to extended star-like configurations, with sets of unconnected nodes that are tethered to the central RC node via reciprocal connections. Such star-like motifs are notable features of network connectivity, since they represent subgraphs with zero clustering and maximally centralized communication through the star's hub node. Three examples of star-like motifs around members of RC1 are shown in Figure 6C, extracted by a heuristic search algorithm that attempted to identify the largest star subgraph centered on each node. On average, star-like motifs surrounding RC nodes were significantly larger than those surrounding non-RC nodes (RC1 medians 9 versus 2; RC2 medians 6 versus 2; both p<0.001, Wilcoxon one-sided rank sum test), and stars on high-degree nodes in degree-conserving randomized networks were significantly reduced in size (RC1 median 5.43±0.54; RC2 median 4.07±0.26; averaged over 200 randomized networks, both p<0.005, one-sided). As might be predicted on the basis of the smaller z-score of motif 9 for latticized compared to randomized networks (Figure 6A), stars in degree-conserving latticized networks were larger than those of randomized controls, but again tended to be smaller than those seen in macaque cortex (RC1 median 7.50±0.71, p<0.05; RC2 median 5.37±0.47, p = 0.31, n.s.; averaged over 200 latticized networks). This result suggests that a combination of high-degree nodes and cost-efficient wiring can partially account for the appearance of large star motifs in macaque cortex. An important difference between macaque cortex and the two null models considered here is that rich club organization, present only in the macaque network, promotes dense structural connectivity among the star's high-degree central nodes.

### Rich Club Paths and Communication Cost

All pairs of nodes in the macaque cortical network are linked by at least one path, and the minimal number of steps needed to travel between two nodes defines their topological distance. The diameter of the graph (the maximal distance between any two nodes) was 5. Across the whole network a total of 479,036 minimally short paths (from now on simply referred to as “paths”) were traced. Note that this number exceeded the number of connected node pairs (58,322) since short paths in binary networks are often non-unique, offering many degenerate solutions. Of all short paths, 444,301 connected pairs of non-rich club nodes outside of RC1 (RC2: 373,632). Of the paths connecting non-RC1 nodes, a large proportion (58.4%) touched at least one RC1 node and 12.1% traveled across at least one RC1 edge (a connection between two RC1 nodes). The corresponding proportions for non-RC2 paths were 87.6% and 41.6%, respectively. To determine the role of node degree in generating these high proportions we performed a comparison to paths found in 200 randomized networks with preserved degree sequence (equal in-degree and out-degree at each node) but degraded global connectivity without rich club organization. For node sets corresponding in degree to those comprising RC1 and RC2 in the macaque, we found proportions of 49.9%±1.0% (touching RC1 nodes), 6.52%±0.6% (traveling RC1 edges), 81.1%±0.7% (touching RC2 nodes), and 28.4%±0.9% (traveling RC2 edges). All of these proportions were significantly (p<0.001) below those found in the macaque cortex, and the effect size was particularly large for paths that travel across rich club edges. This comparison suggests that the high proportion of paths that involved either rich club nodes or rich club edges was only partly due to their high degree, and that rich club organization significantly adds to the effect.

Even higher proportions were encountered in an analysis of rich-club participation in paths that connect nodes across different modules. Of all paths that cross between modules, 61% involved at least one RC1 node, and 90% involved at least one RC2 node, reaching as high as 97% for specific module pairs. Finally, the number of paths in which each edge participates was significantly higher for both rich club connections (RC1 median = 478; RC2 median = 244) and feeder connections (RC1 median = 389; RC2 median = 266) compared to local connections (RC1 median = 190; RC2 median = 134; all p<0.001, Wilcoxon one-sided rank sum test). Taken together, these results suggest that rich club nodes and connections made a strong contribution to paths in the macaque cortex, particularly those linking nodes in different network communities, a contribution which is stronger than that predicted by rich club size or density (RC1 accounts for 5% of all nodes and 2% of all connections; RC2: 14% and 12%, respectively).

**Figure 7:**
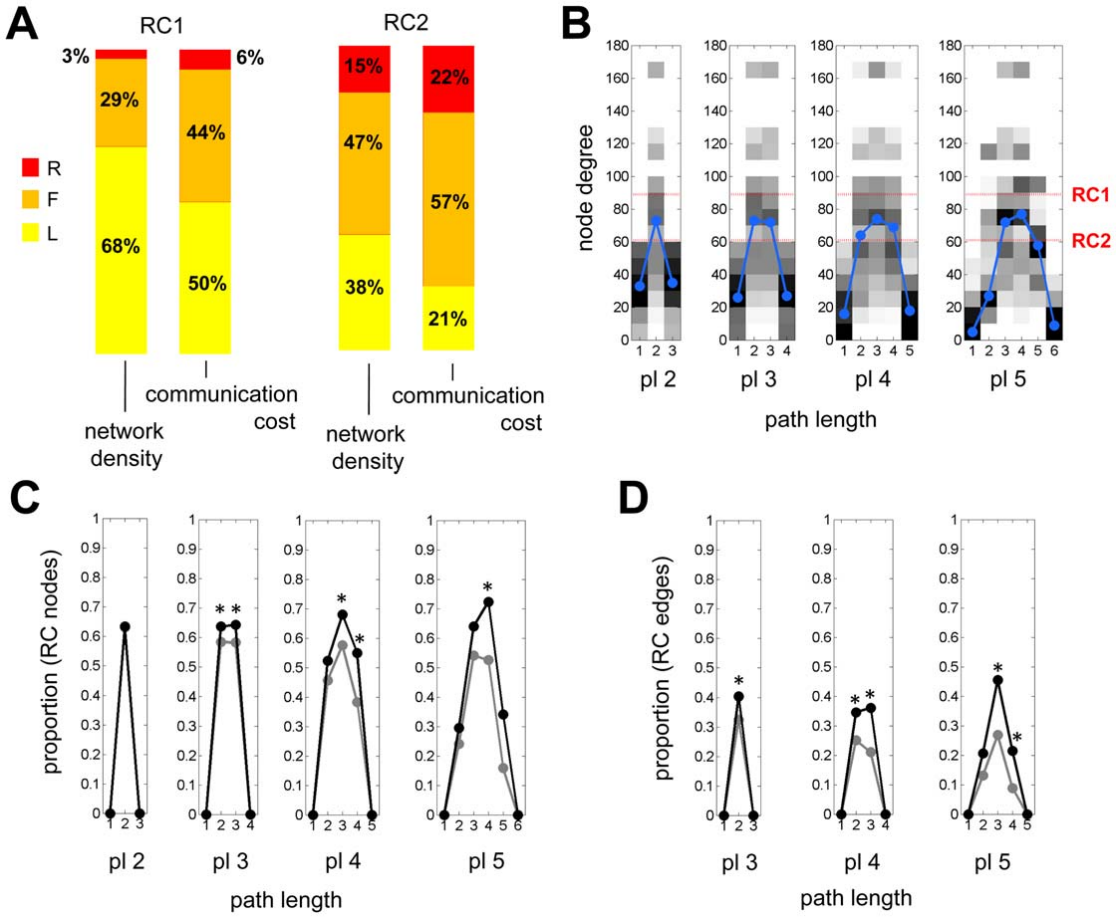
Communication cost and path structure. (A) Bar plots display the proportions of rich club (“R”, red), feeder (“F”, orange) and local (“L”, yellow) connections to network density and communication cost, shown for RC1 and RC2. (B) Heatmap (gray scale) of node degrees encountered along paths of path length (pl) 2, 3, 4, and 5. The median degrees are indicated in blue, and minimum degrees for nodes that are members of RC1 and RC2 are shown in red. The plot aggregates data on all paths starting and ending on non-rich club (non-RC2) nodes. Similar plots are obtained for paths starting and ending on non-RC1 nodes (not shown). (C) Proportion (probability) of touching an RC2 node plotted along all paths of lengths 2 to 5 steps. (D) Proportion (probability) of traveling along an RC2 edge plotted along all paths of lengths 3 to 5 steps. In panels (C) and (D), black symbols represent data from the macaque network, gray symbols represent means from 200 randomized networks preserving node degree sequence. Asterisks indicate that macaque data exceed the values from the empirical null distributions at p<0.05 (one-sided, uncorrected).

A previous study of human brain connectivity demonstrated that rich club connections tend to span longer distances, participate in a large proportion of all shortest communication paths and account for a large proportion of the network's communication cost [24]. In agreement with these findings, statistical comparisons carried out for both RC1 and RC2 in the macaque cortex revealed that rich club and feeder connections span longer Euclidean distances than local connections (both p<0.001, Wilcoxon one-sided rank sum test). Concordantly, the communication cost per edge, defined here as the product of the number of paths to which each edge contributes and the edge's physical length (approximated as inter-nodal Euclidean distance), was significantly greater for rich club and feeder connections than for local connections (both p<0.001, Wilcoxon one-sided rank sum test). Figure 7A gives a graphical comparison of the proportional contributions of rich club, feeder and local connections to network density and communication cost, computed for only those 187 nodes (and their connections) for which spatial coordinates were available. For both RCs, only a small proportion of the network density was accounted for by rich club connections, while the same connections accounted for a much larger proportion (×2.41 for RC1, ×1.42 for RC2) of the communication cost. Thus, computing the proportions of the communication cost for rich club, feeder and local connections across the whole network suggested that rich club connections account for a large proportion of the communication cost. Care must be taken when interpreting these results, since data on actual fiber trajectories and tract volumes were missing from the analysis and Euclidean distances were used instead to estimate wiring length.

The high participation of rich club nodes and connections in paths across the network reflects a propensity of the rich club to attract and disperse traffic routes for global brain communication. An analysis of the node degrees encountered along paths between non-rich club nodes provided further insight into this phenomenon [24]. Figure 7B shows data on node degree as a function of path length, for all paths of lengths 2, 3, 4 and 5 steps linking non-RC2 nodes. The plots were generated by creating a 2D histogram for each path length, displayed here as a gray scale “heatmap”, that summarizes the distributions of node degrees encountered along the path, stepping from the source to the target node. In all plots, the median node degree at first increases, approaching high levels as more and more paths touch on or travel among rich club nodes, and then decreases as paths terminate on the target node. Consistently, the probability of touching a rich club node or traveling on a rich club edge was highest near the middle of each path, and significantly exceeded the corresponding probabilities encountered in degree-preserved randomized networks (Figure 7C,D).

### Rich Club and Directionality of Connections and Paths

**Figure 8:**
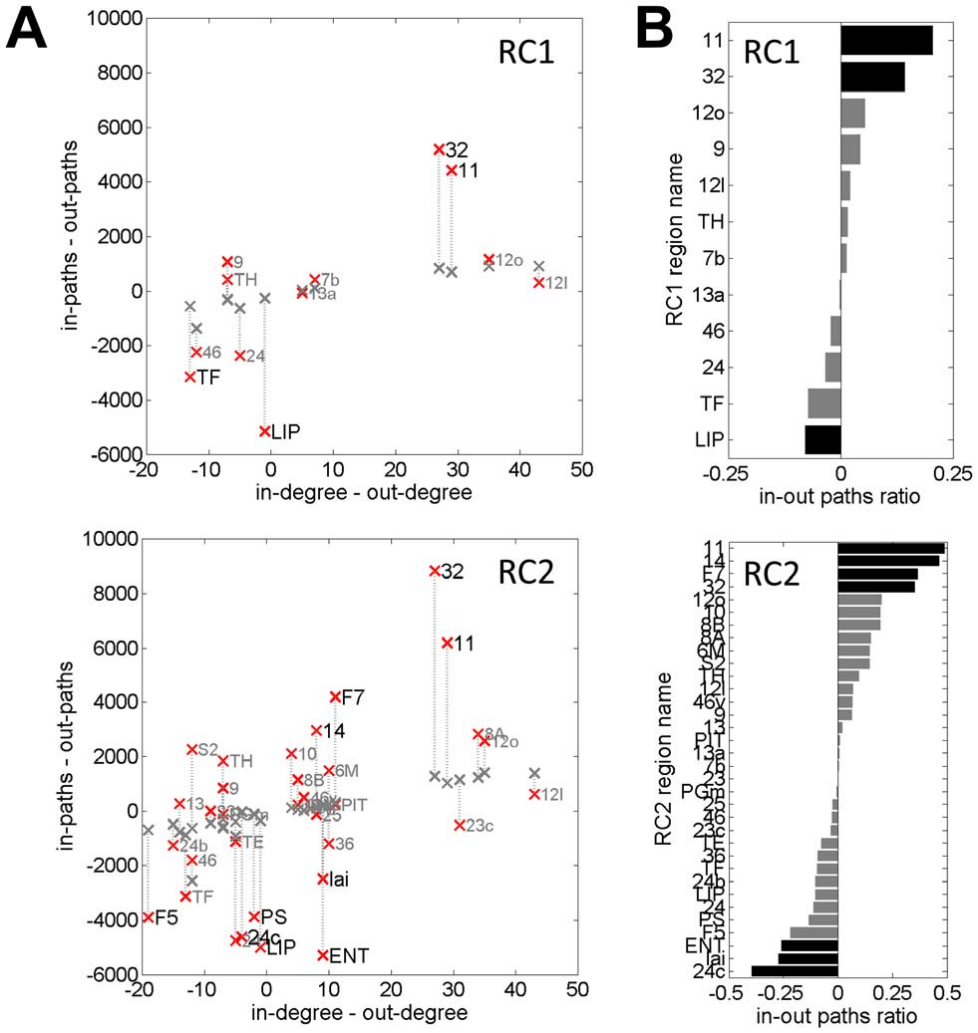
Directionality of paths and rich club. The figure presents distributions of short communication paths leading into (“in-paths”) and emerging from (“out-paths”) rich club nodes. Plots at the top are for RC1 regions, and plots at the bottom are for RC2 regions. Data shown are for paths that begin and end on non-RC nodes. (A) Scatter plot of degree imbalance (in-degree minus out-degree) and path imbalance (in-paths minus out-paths) for the macaque cortex (red markers) and for averages obtained from 200 randomized networks preserving degree sequence (gray markers). Note that node degree imbalances are identical for randomized networks. Region names in large black font mark regions for which the difference between macaque and random population is significant (p<0.01, uncorrected). (B) The in-out paths ratio, shown here for the macaque cortex, is computed as (in-paths−out-paths)/(in-paths+out-paths). Black bars indicate regions for which the in-out paths ratio is significantly different (p<0.01, uncorrected) compared to that of a population of 200 randomized networks.

Tract tracing data delivers information on the direction of projections and thus offers the opportunity to determine the directions of paths as they enter and leave the rich club at specific nodes, an advantage compared to present-day diffusion imaging approaches. As previously reported, many cortical regions maintain an unequal number of afferent and efferent anatomical projections, rendering some nodes net-emitters (“sources”) and others net-receivers (“sinks”) [54]. Figure 8 shows scatter plots of the balance of in-degree and out-degree versus the number of paths (starting and ending on non-RC nodes) that enter (“in-paths”) or leave (“out-paths”) the rich club, at each rich club node comprising RC1 (Figure 8, top) and RC2 (Figure 8, bottom). As expected, the two measures were moderately correlated (R = 0.62 at p = 0.03, and R = 0.49 at p = 0.003 for RC1 and RC2, respectively). To account for the effect introduced by the in-/out-degree distribution, we performed a comparison of the macaque network to a population of 200 randomized networks with preserved degree sequence (equal in-degree and out-degree at each node) but degraded global connectivity without rich club organization. Statistical comparison revealed several RC regions that exhibit significant (p<0.01, uncorrected) asymmetry in the number of incoming or outgoing paths, either expressed as a total count (Figure 8A) or as a proportion (Figure 8B). In RC2, regions 11, 14, F7 and 32 of the macaque cortex absorbed at least 25% more paths into the rich club than they emit, while regions ENT, Iai and 24c exhibited a greater than 25% excess in outgoing paths, and these imbalances significantly exceeded those seen in randomized networks preserving all node degrees and degree imbalances. These anatomical patterns of directionality of paths suggest that some regions of the rich club are specialized for collecting signals traveling along short paths, while others are specialized for emitting signals via short paths onto target regions.

## Discussion

A graph-theoretical analysis of a newly collated connectivity matrix of the macaque cerebral cortex revealed the existence of 5 network communities that were cross-linked by a rich club consisting of between 12 and 45 densely interconnected high-degree brain regions. Many rich club members were identified as connector hubs, and were highly ranked across a range of centrality measures. Rich club regions were found to participate in extended star-like motifs, where they were preferentially located at the star's center linking diverse sets of regions across all structural communities. Other network attributes strongly suggested a central role of the rich club for brain communication along short paths. A large proportion of such paths touched or traveled through the rich club, with rich club connections making a contribution to the network's overall communication cost that is greater than predicted by their density alone. Many short paths exhibit a characteristic degree structure. Paths first travel across nodes of, on average, increasing degree, with many of them reaching rich club nodes, and then move across nodes of lower degree towards their targets. Furthermore, an analysis of the directionality of paths into and out of the rich club reveals that some nodes appear specialized for attracting and others for dispersing signals traveling along short paths. Overall, our study finds evidence for robust rich club organization in the cortex of a non-human primate, demonstrated in a large-scale network constructed from tract tracing data. Here, as in the human brain [23], [24], the rich club's placement within the cortical architecture strongly suggests a central role in brain global communication, and in integrating information across segregated network communities.

Several of the features of macaque network organization identified in this report are consistent with findings in other mammalian species, including the cat cortex [22] and the human cortex [23], [24]. These features include robust small-world attributes, and a pronounced community structure with modules that are interlinked by a set of connector hubs and high centrality nodes including widely dispersed multimodal regions in prefrontal, parietal and temporal cortex. In the present report, a statistical analysis of the density of interconnections among high-degree brain regions provided evidence for the existence of a rich club in the macaque cortex. The majority of rich club regions can be characterized as multimodal or transmodal areas [55] whose constituent neurons show complex physiological responses related to social communication, executive control, spatial attention, reward processing, or episodic memory. This composition of the macaque rich club is consistent with the complex physiological characteristics of rich club regions identified in human cortex [23]. While caution is necessary when inferring homology of brain systems across species [56] the present analysis does suggest that overlapping sets of regions in macaque and human brain share the common topological attribute of being highly and densely interconnected, forming an integrative network core. Based solely on this shared attribute, the members of the rich club found in the human and macaque cortex comprised portions of the superior frontal (prefrontal) cortex (macaque areas 9, 46, F5, F7, 8A), precuneus (area PGm, 31), superior parietal cortex (areas 7b, LIP), posterior cingulate cortex (areas 23, 23c), medial orbitofrontal cortex (areas 10, 11, 12o, 12l, 13, 13a and 14), the anterior cingulate cortex (area 24), the entorhinal (perirhinal) cortex (areas ENT, 36), the parahippocampal gyrus (areas TH, TF) and the insula (area Iai). These common rich club regions are linked by connections that can be anatomically related to long association fiber pathways of the primate brain [57], [58], including the superior longitudinal fasciculus (linking, among others, rich club areas 46, 9, F7, PGm, LIP, 7b), the inferior longitudinal fasciculus (areas LIP, TE, TH, TF, PIT), the uncinate fasciculus (areas 10, 11, 13, 32, TE, TH) and the cingulum bundle (areas 14, 25, 32, F7, TF, TH). Their linkage within a densely connected subgraph suggests that these pathways form a coherent system for integration and communication.

Several aspects of the present analysis aimed at characterizing the contributions of the rich club to long-distance global brain communication. Four main results stood out in this regard. First, confirming earlier reports on hubs in the cat cortex [18], [22] and the human cortex [23], [24], the macaque rich club was found to cross-link structural communities rather than representing a segregated subgraph or module of its own. Rich club regions formed a collective of connector hubs, with high incidences of apex motifs and star-like configurations that straddled module boundaries. This topological arrangement is conducive to a central role in inter-module communication, involving not only the creation of short communication paths, but also the integration of specialized (segregated) information. Our findings support the conclusions of earlier work [6], [22] suggesting that a key role of rich club organization is to collect segregated information and combine it in a manner that promotes adaptive function, thus serving to balance functional segregation and integration [59], [60]. Second, rich club nodes and connections participated in a disproportionately large number of short paths linking regions all across the brain. The proportion was highest for paths that crossed module boundaries. This topological layout suggests a propensity of the rich club to attract long-distance signal traffic, again commensurate with a central role in integrative processing [23]. Third, the existence of a highly connected collective of rich club regions entailed that short paths tended to follow a distinct pattern of traveling across nodes of first increasing and then decreasing degree. Presence of rich club organization was associated with high probabilities that paths touched on rich club nodes or traveled on rich club edges, exceeding those observed in degree-matched null models. These results are in accordance with the prevalence of ascending/descending degree along paths in human cortical networks derived from diffusion imaging and tractography [24]. The degree structure of short paths suggests that it may facilitate efficient strategies for long-distance navigation that take advantage of local information, rather than requiring global knowledge about the brain's connection topology [61]. Fourth, the availability of directional information on individual connections allowed an investigation of the flow of communication paths into and out of specific rich club regions. It appears from this analysis that some of the rich club regions located in the prefrontal/frontal cortex (areas 11, 14, F7, 32) collect a greater number of paths than they emit, with regions in anterior cingulate, temporal and insular cortex (areas 24c, ENT, Iai) exhibiting the opposite effect. This suggests that the net flow of neural communication traverses the rich club in a specific direction, along patterns imposed by asymmetric non-reciprocal projections. Such projections have been consistently reported in macaque tract tracing data, including recent quantitative labeling studies [62].

An important aspect of the present study is that the main results regarding the role of the rich club in macaque brain communication are broadly consistent with findings reported for the human brain [24], despite significant differences in data collection methods (tract tracing yielding binary and directed pathways in the macaque versus tractography yielding weighted and undirected fiber tracts in human). This suggests that the rich club is a robustly detected feature of large-scale brain architecture, and inferences about its potential role in communication processes, at least up to a point, do not depend on the methodology used for measuring anatomy. The results presented in this report await further refinement and elaboration, particularly through the acquisition of more comprehensive and quantitative connectivity data sets, from the macro to the micro level, in non-human primates (e.g. [62]) and other mammalian species (e.g. [63], [64]). Especially important will be the inclusion of quantitative tract tracing data recording the density of inter-regional projections, now known to range over several orders of magnitude in both macaque [62] and mouse brain [65]. The inclusion of quantitative data on projection density obtained from tract tracing is especially important as diffusion imaging and tractography tend to under-represent weak or sparse projections. Weighted network analysis of community structure, regional centrality and rich club organization, as well as other measures for detecting networks cores [66], will provide more detailed insight into the layout of cortical connections and the short communication paths they support. Increased spatial resolution in labeling and tracing of axonal projections among rich club regions will be essential to possibly discern distributed parallel circuits [67], and to clarify if synaptic links among highly connected regions are made by common populations of neurons with branching axonal collaterals or by distinct populations of projection neurons [68].

The current data set [27] imposes several important limitations that need to be taken into account when interpreting our results. First, the connection matrix is based on a hierarchical matrix of brain regions, collected from numerous tract tracing studies, and thus does not incorporate a single parcellation based on a consistent set of anatomical and/or physiological criteria. This fact results in some redundancies of pathways that are reported in different studies for overlapping or nested sets of regions. For example, the connection matrix contains entries for cortical region 10 while also recording connectivity for substructures 10m, 10v, 10o and 10d. Modularity analysis places regions 10m, 10v and 10o into module 1, while regions 10d as well as the hierarchically higher region 10 (which contains all substructures) are placed into module 3. Additional conflicts arise since different parcellations may refer to a given region by the same anatomical name, but may assign the region somewhat different spatial extent and location. These limitations can be overcome through improved strategies for spatial registration and alignment of connectivity data across parcellations [69], as well as new measurements of anatomical projections with the goal of characterizing inter-regional connectivity uniformly and quantitatively [62].

A second significant limitation of the current data set is that it does not contain measurements on the trajectories and volumes of fiber tracts, thus precluding a detailed assessment of the connection cost incurred by dense rich club connectivity. In the present study, substituting Euclidean distances between connected region pairs as a proxy for fiber lengths suggested that, as shown previously in human diffusion imaging data [24], rich club and feeder connections tend to span longer distances than local connections and account for a large proportion of the brain's communication cost. However, more refined analyses are clearly needed to further investigate whether the rich club is a high-cost feature of the primate brain, thus representing an example for the economic tradeoff between cost and efficiency that drives brain network organization [70].

We hypothesize that the existence of a rich club may have far-reaching consequences for brain dynamics and communication processes. The connection topology of a central collective of densely interconnected regions that attract and disperse large volumes of signal traffic is reminiscent of proposals of an integrative core or “workspace” in modern theories of cognition and consciousness [71]–[75]. If the global network of structural brain connectivity, the connectome [76], [77], is indeed important for shaping the dynamic flow of information across the brain, the aggregation of highly connected brain regions into a core or rich club may prove to be a crucial anatomical substrate for the extensive repertoire of integrative cognitive processes encountered in highly evolved brains.

## Supporting Information

File S1 **Connection matrix.**
(MAT)

File S2 **Region names.**
(TXT)
